# Novel mutations in *RPE65* identified in consanguineous Pakistani families with retinal dystrophy

**Published:** 2013-07-19

**Authors:** Firoz Kabir, Shagufta Naz, S. Amer Riazuddin, Muhammad Asif Naeem, Shaheen N. Khan, Tayyab Husnain, Javed Akram, Paul A. Sieving, J. Fielding Hejtmancik, Sheikh Riazuddin

**Affiliations:** 1 National Centre of Excellence in Molecular Biology, University of the Punjab, Lahore, Pakistan; 2 The Wilmer Eye Institute, Johns Hopkins University School of Medicine, Baltimore MD; 3 Allama Iqbal Medical College, University of Health Sciences, Lahore, Pakistan; 4 Ophthalmic Genetics and Visual Function Branch, National Eye Institute, National Institutes of Health, Bethesda, MD

## Abstract

**Purpose:**

To identify pathogenic mutations responsible for retinal dystrophy in three consanguineous Pakistani families.

**Methods:**

A thorough ophthalmic examination including fundus examination and electroretinography was performed, and blood samples were collected from all participating members. Genomic DNA was extracted, and genome-wide linkage and/or exclusion analyses were completed with fluorescently labeled short tandem repeat microsatellite markers. Two-point Lod scores were calculated, and coding exons along with exon-intron boundaries of *RPE65* gene were sequenced, bidirectionally.

**Results:**

Ophthalmic examinations of the patients affected in all three families suggested retinal dystrophy with an early, most probably congenital, onset. Genome-wide linkage and/or exclusion analyses localized the critical interval in all three families to chromosome 1p31 harboring *RPE65*. Bidirectional sequencing of *RPE65* identified a splice acceptor site variation in intron 2: c.95–1G>A, a single base substitution in exon 3: c.179T>C, and a single base deletion in exon 5: c.361delT in the three families, respectively. All three variations segregated with the disease phenotype in their respective families and were absent from ethnically matched control chromosomes.

**Conclusions:**

These results strongly suggest that causal mutations in *RPE65* are responsible for retinal dystrophy in the affected individuals of these consanguineous Pakistani families.

## Introduction

Retinal dystrophies are debilitating disorders of visual function that primarily affect the ocular retina. Among the inherited retinal dystrophies, retinitis pigmentosa (RP; OMIM 268000) contributes significantly to the total number of cases of blindness worldwide [1]. RP primarily affects the rod photoreceptors whereas the function of the cone receptors is compromised as the disease progresses [2,3]. Clinical symptoms include appearance of melanin-containing structures in the retinal vascular layer, attenuated arterioles, and bone spicule-like pigmentation in the fundi [2,3]. Affected individuals often have severely abnormal or non-detectable electroretinographic responses even in the early stage of the disease [2,3].

The RPE65 protein was first described by Hamel and colleagues [4]. It is involved in many aspects of retinal metabolism that are essential for maintaining the photoreceptor cells [5,6]. *RPE65* consists of 14 coding exons spanning a 20 kb region and encodes for a 533 amino acid protein [7]. RPE65 is an abundant protein in the retinal pigment epithelium (RPE) and is associated with the microsomal membrane [4,8]. More than 60 different mutations in the *RPE65* gene have been associated with inherited retinal dystrophies including Leber congenital amaurosis (LCA) and autosomal recessive RP [9,10]. Mutations in the *RPE65* gene account for approximately 2% of the total genetic load of recessive RP and approximately 16% of LCA [11-13].

Several animal models mimicking the *RPE6*5 mutations have been developed and characterized. Among these, *Rpe65* homozygous knockout mice develop slow retinal degeneration that results in loss of 30% of the photoreceptors by 12 months of age [14]. The severity of the disease phenotype increases with time resulting in more severe photoreceptor loss at 24 months [14]. The *Rpe65* knockout mice lack 11-*cis* retinal and 11-*cis*-retinyl esters, and accumulate excessive levels of all-*trans* retinyl esters in the RPE, which supports the notion that RPE65 is essential for the isomerization of all-*trans* retinyl esters [14,15].

Here, we report three highly inbred families with multiple consanguineous marriages diagnosed with early onset of RP. Genome-wide linkage and/or exclusion analyses localized the disease interval to chromosome 1p31 harboring *RPE65*. Bidirectional sequencing of *RPE65* identified a splice acceptor site variation in intron 2: c.95–1G>A, a single base substitution in exon 3: c.179T>C, and single base deletion in exon 5: c.361delT in these three families, respectively. All three variations segregated with the disease phenotype in their respective families and were absent from ethnically matched control chromosomes.

## Methods

### Clinical ascertainment

One hundred and twenty-five consanguineous Pakistani families with autosomal recessive retinitis pigmentosa were recruited to participate in a collaborative study between the National Centre of Excellence in Molecular Biology (NCEMB), Lahore, Pakistan, and the National Eye Institute (NEI), Bethesda, Maryland, to identify new disease loci and genes. Institutional Review Board (IRB) approval was obtained for this study from both institutes. The participating subjects gave informed consent consistent with the tenets of the Declaration of Helsinki. All three families described in this study are from the Punjab province of Pakistan. A detailed medical history was obtained by interviewing family members. Funduscopy was performed at Layton Rehmatulla Benevolent Trust (LRBT) Hospital, Lahore. Electroretinography measurements were recorded using equipment manufactured by LKC (Gaithersburg, MD) and performed according to the standards of the International Society for Clinical Electrophysiology (ISCEV) [16]. Rod-cone response was measured at 0 dB, whereas isolated cone responses were recorded at 0 dB with a 30 Hz flicker to a background illumination of 17–34 cd/m^2^. Blood samples were collected from affected and unaffected family members, and genomic DNA was extracted with a non-organic method as described previously [17,18].

### Genotype analysis

Genome-wide and exclusion analyses were performed with highly polymorphic fluorescent markers in multiplex PCR reactions. Briefly, each reaction was performed in a 5 μl mixture containing 40 ng genomic DNA, various combinations of 10 μM-dye-labeled primer pairs, 1X GeneAmp PCR buffer, 1 mM deoxynucleotide triphosphate mix, 2.5 mM MgCl_2_, and 0.2 U Taq DNA polymerase. Initial denaturation was performed for 5 min at 95 °C, followed by 10 cycles of 15 s at 94 °C, 15 s at 55 °C and 30 s at 72 °C, and then 20 cycles of 15 s at 89 °C, 15 s at 55 °C, and 30 s at 72 °C. The final extension was performed for 10 min at 72 °C and followed by a final hold at 4 °C. PCR products from each DNA sample were pooled and mixed with HD-400 size standards (Applied Biosystems, Foster City, CA). The resulting PCR products were separated in an ABI 3100 DNA analyzer, and alleles were assigned using GeneMapper Software version 4.0 (Applied Biosystems).

### Linkage analysis

Two-point linkage analyses were performed using the FASTLINK version of MLINK from the LINKAGE Program Package [19,20]. Maximum LOD scores were calculated using ILINK. Autosomal recessive retinitis pigmentosa was analyzed as a fully penetrant trait with an affected allele frequency of 0.001. The marker order and the distances between the markers were obtained from the Généthon database and the NCBI chromosome 1 sequence maps. Allele frequencies were estimated from 96 unrelated and unaffected individuals from the Punjab province of Pakistan.

### Mutation screening

Primer pairs for individual exons were designed using the primer3 software (Table 1). Amplifications were performed in a 25 μl reaction containing 50 ng genomic DNA, 8 pmol each primer, 250 μM deoxynucleotide triphosphate, 2.5 mM MgCl_2_, and 0.2 U Taq DNA polymerase in the standard 1X PCR buffer provided by the manufacturer (Applied Biosystems). PCR amplification consisted of a denaturation step at 96 °C for 5 min, followed by 40 cycles, each consisting of 96 °C for 45 s followed by 57 °C (or the specific annealing temperature of the primer pair) for 45 s and at 72 °C for 1 min. PCR products were analyzed on 2% agarose gel, precipitated and purified by ethanol precipitation. The PCR primers for each exon were used for bidirectional sequencing using the Big Dye Terminator Ready reaction mix according to the manufacturer’s instructions (Applied Biosystems). Sequencing products were resuspended in 10 μl formamide (Applied Biosystems) and denatured at 95 °C for 5 min. Sequencing was performed on an ABI 3100 DNA analyzer (Applied Biosystems). Sequencing results were assembled using ABI PRISM sequencing analysis software version 3.7 and analyzed using SeqScape software (Applied Biosystems).

**Table 1:** Primer sequences used for bi-directionally sequencing of *RPE65*. The amplification conditions are described in the methods and materials.

**Exon**	**Forward Primer**	**Reverse Primer**	**Annealing Temperatures (°C)**
**1**	GAGAGCTGAAAGCAACTTCTG	ATAGCACATTTATCATGAATCCATG	54
**2**	CTATCTTGCGGACTTTGAGC	GCCAGAGAAGAGAGACTGAC	60
**3**	GGCAGGGATAAGAAGCAATG	CTGAGTTCAGAGGTGAAAAC	63
**4–5**	CTGTACGGATTGCTCCTGTC	TTAGAATCATACATTCGCAGCATG	57
**6**	TATAATGTATCTTCCTTCTCTCAAC	CTCACAATACAGTAACTTTCTCAC	57
**7–8**	AAATAAGAGGCTGTTCCAAAGC	TTAAACACATCTTCTTCAGAATCAC	54
**9**	GTACACTTTTTTCCTTTTTAAATGCATC	GTTTTAGATGTGATTCAGATTGAGTG	57
**10**	TTGTCATTGCCTGTGCTCATG	TGAGAGAGATGAAACATTCTGG	57
**11**	TCTGCATTTCTGGCTGTTTG	AAGTGATTCAAGCCAAGTCCA	55
**12–13**	CACACGGGAGTGAACAAATG	CAACCTTACTCCTTTCCTAACGA	55
**14**	AGTCAGAAAAAGAAGTCAGGTC	ATTGCTTGCTCAACTCAGTGC	60

### Prediction analysis

Evolutionary conservation of the L60 in other *RPE65* orthologs was examined using the UCSC genome browser. The possible impact of an amino acid substitution on the structure of *RPE65* was examined with SIFT and PolyPhen tools available online [21,22].

## Results

**Figure 1:**
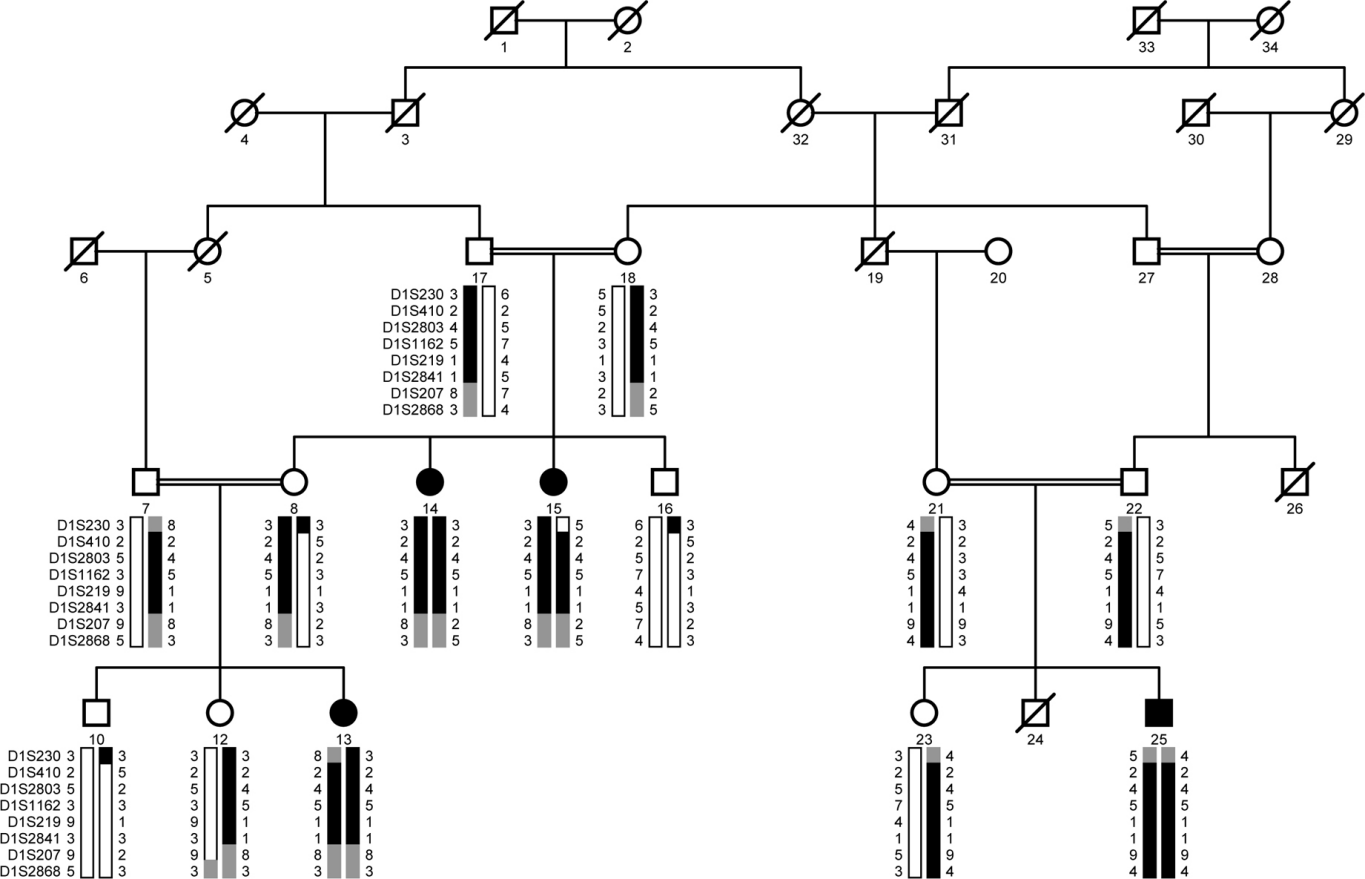
Pedigree drawing of family PKRP020 with haplotypes of chromosome 1p markers. Squares represent men, circles represent women, filled symbols are affected individuals, a double line between individuals indicates consanguinity, and a diagonal line through a symbol is a deceased family member. The haplotypes of eight adjacent chromosome 1p31 microsatellite markers are shown with alleles forming the risk haplotype shaded black, alleles cosegregating with retinal dystrophy but not showing homozygosity shaded gray, and alleles not cosegregating with retinal dystrophy are shown in white.

**Figure 2:**
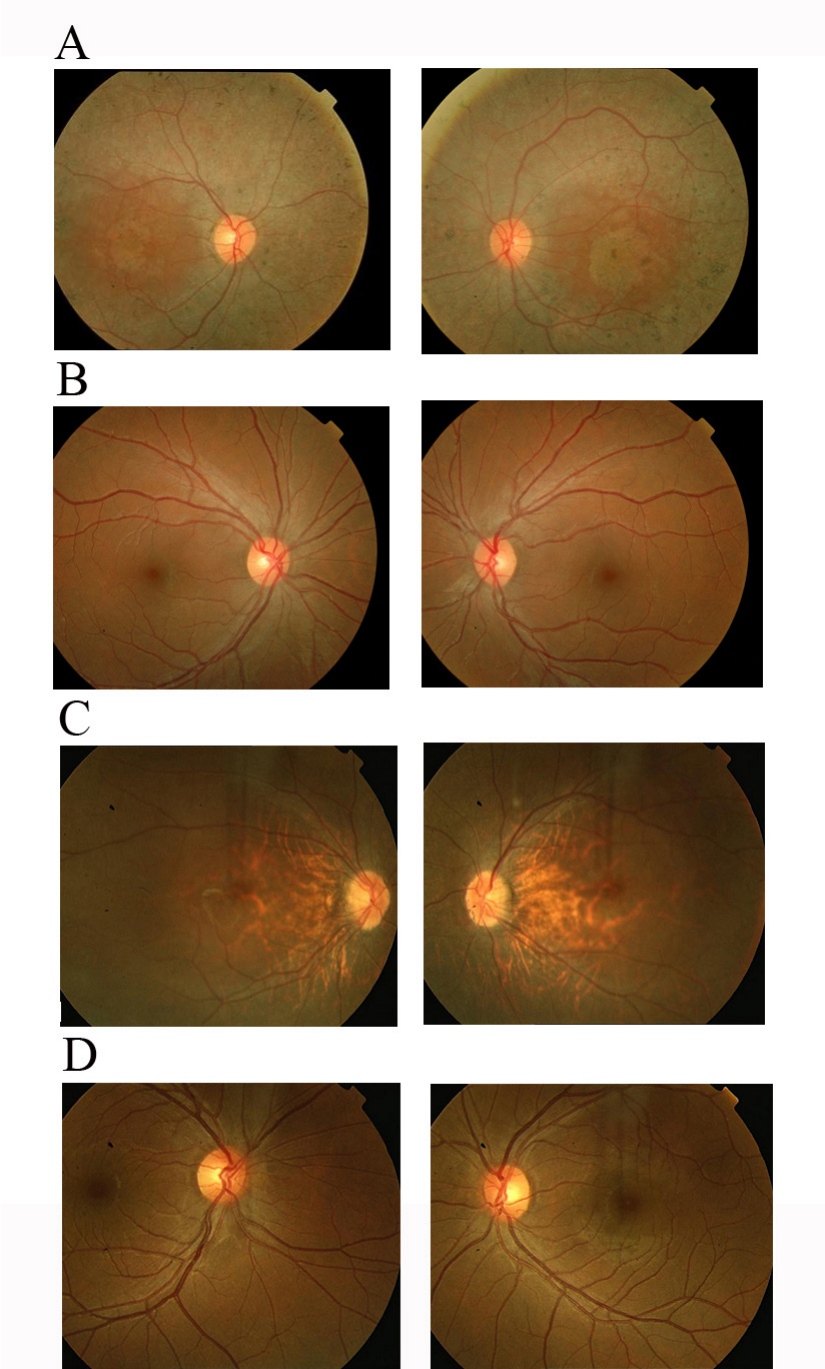
Fundus photographs of affected individuals of families PKRP020 and PKRP160. **A**: OD: right eye, and OS: left eye: affected individual 13 and **B**: OD and OS: unaffected individual 16 of PKRP020. **C**: OD and OS: affected individual 9 and **D**: OD and OS: unaffected individual 10 of PKRP160. Fundus photographs of affected individuals show peripheral fundus demonstrating several features associated with retinitis pigmentosa (RP) including a waxy pallor of the optic disc, attenuated arterioles, and peripheral bone spicules.

**Figure 3:**
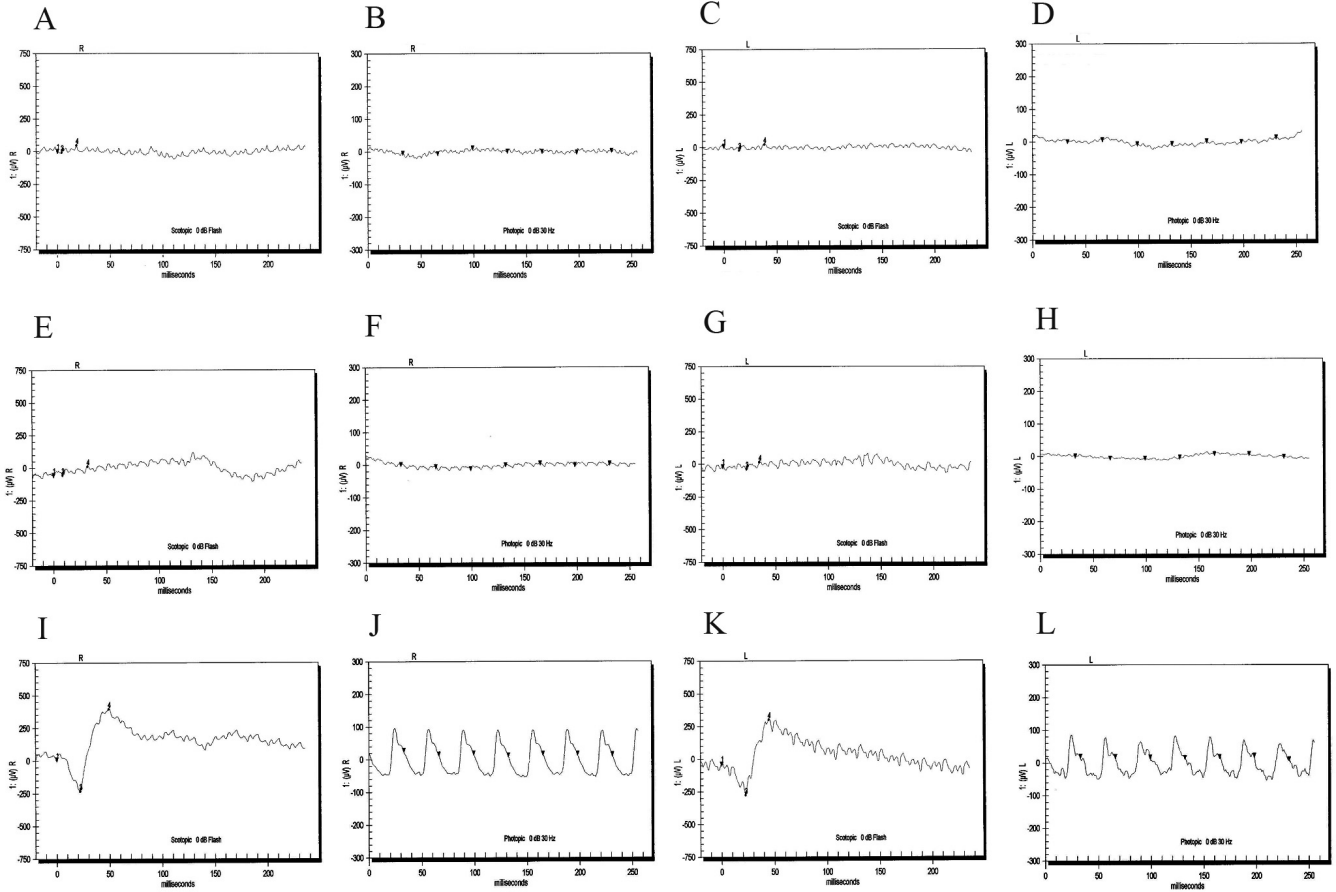
Electroretinography recordings of PKRP020 and PKRP160 family members. **A**: OD: right eye, combined rod and cone response. **B**: OD cone flicker response. **C**: OS: left eye, combined rod and cone response. **D**: OS cone flicker response of affected individual 13 of PKRP020. **E**: OD combined rod and cone response. **F**: OD cone flicker response. **G**: OS combined rod and cone response. **H**: OS cone flicker response of affected individual 9 in PKRP160. I: OD combined rod and cone response. **J**: OD cone flicker response. **K**: OS combined rod and cone response. **L**: OS cone flicker response of unaffected individual 10 of PKRP160. The affected individuals have typical retinitis pigmentosa (RP) changes including loss of rod and cone responses.

Family PKRP020 is from the Punjab province of Pakistan (Figure 1). A detailed medical history was obtained by interviewing family members. All patients have experienced night blindness and constriction of peripheral visual field since the early years of their lives. Affected individuals showed bone spicule-like formation in the fundus, attenuation of the retinal arterioles, and pallor of the optic disc (Figure 2A). The electroretinographic responses were non-detectable suggesting advanced stage of rod-cone dystrophy (Figure 3A–D). Taken together, the ophthalmological examinations show typical features of retinal dystrophies and fulfill the diagnostic criteria of RP. However, given the uncertainty of the age of onset, we cannot conclusively differentiate between LCA and autosomal recessive RP.

A genome-wide scan was completed with 382 short tandem repeat (STR) markers spanning the entire human genome at approximately 10 cM spacing. During the genome-wide scan, evidence of linkage for PKRP020 was observed with adjacent marker markers on chromosome 1. A maximum two-point LOD score of 4.44 was obtained with marker D1S1162 at θ=0 (Table 2A). Subsequently, additional STR markers were selected from the Généthon and Marshfield databases. Two-point LOD scores of 2.50, 4.37, 2.92,, and 2.92 were obtained with markers D1S410, D1S2803, D1S219, and D1S2841 at θ=0, respectively (Table 2A).

**Table 2:** Two point lod scores of chromosome 1p markers for families A) PKRP020, B) PKRP160, and C) PKRP235.

**A**											
Marker	cM	Mb	0	0.01	0.05	0.09	0.1	0.2	0.3	Z**_max_**	Ɵ**_max_**
D1S230*	95.31	62.6	- ∞	-5.1	-2.24	-1.11	-1.11	-0.25	0	0.2	0.4
D1S410	100.4	68.13	2.5	2.44	2.22	1.94	1.90	1.36	0.79	2.5	0
D1S2803	101.5	68.91	4.37	4.28	3.9	3.42	3.4	2.41	1.39	4.37	0
D1S1162*	102	69.44	4.44	4.35	3.98	3.5	3.47	2.5	1.49	4.44	0
D1S219	101.5	69.84	2.92	2.85	2.58	2.24	2.22	1.55	0.9	2.92	0
D1S2841	106.4	79.48	2.92	2.85	2.58	2.24	2.2	1.55	0.9	2.92	0
D1S207*	113.69	82.54	-5.22	-0.54	0.6	0.88	0.85	0.84	0.55	0.91	0.13
D1S2868*	126.16	93.33	- ∞	-1.9	-0.14	0.35	0.45	0.48	0.33	0.49	0.17
**B**											
Marker	cM	Mb	0	0.01	0.05	0.09	0.1	0.2	0.3	Z**_max_**	Ɵ**_max_**
D1S198	99.3	67.01	1.97	1.93	1.77	1.61	1.57	1.15	74	1.97	0
D1S1162	102	69.44	3.04	2.98	2.76	2.53	2.47	1.86	1.22	3.04	0
D1S2841	106.4	79.48	3.04	2.98	2.76	2.53	2.47	1.86	1.22	3.04	0
D1S2865	120.28	88.35	-2.14	-0.74	-0.15	-0.01	0	0.01	-0.06	0.01	0.2
**C**											
Marker	cM	Mb	0	0.01	0.05	0.09	0.1	0.2	0.3	Z**_max_**	Ɵ**_max_**
D1S198	99.3	67.01	2.5	2.44	2.22	1.94	1.9	1.36	0.79	2.5	0
D1S2803	101.5	68.91	2.45	2.38	2.14	1.9	1.86	1.27	0.71	2.45	0
D1S1162	102	69.44	2.47	2.4	2.16	1.92	1.87	1.29	0.74	2.47	0
D1S2865	120.28	88.35	2.39	2.31	2.12	1.85	1.81	1.25	0.68	2.39	0

Two-point Lod scores were calculated at different θ values for each marker with the FASTLINK version of MLINK from the LINKAGE program package. Maximum lod scores for each marker were calculated using ILINK. Asterisk indicates markers used in the genome-wide scan.

**Figure 4:**
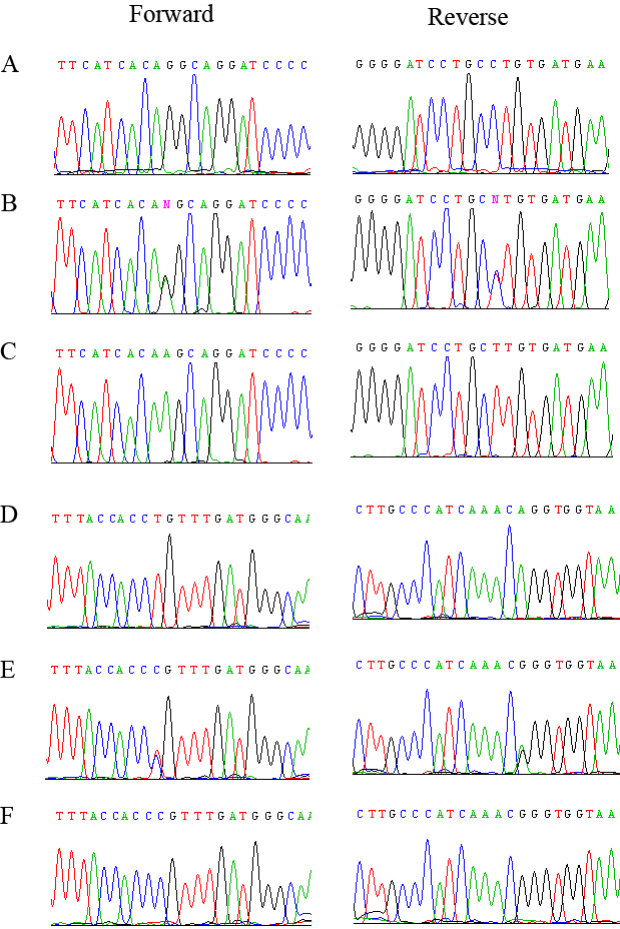
Sequence chromatograms of RPE65 variations identified in families PKRP020 and PKRP0160. **A**: Unaffected individual 10 of family PKRP020 homozygous for the wild-type. **B**: Unaffected individual 12 of family PKRP020 heterozygous carrier and, **C**: affected individual 13 of family PKRP020 homozygous for the G to A transition in intron 2; c.95–1G>A. **D**: Unaffected individual 7 of family PKRP160 homozygous wild-type. **E**: Unaffected individual 6 of family PKRP160 heterozygous carrier and, **F**: affected individual 9 of family PKRP160 homozygous for a T to C transition in exon 3: c.179T>C.

Visual inspection of the haplotypes supports the linkage of PKRP020 to chromosome 1p. As shown in Figure 1, there is a proximal recombination event at D1S230 in affected individual 15 that defines the proximal boundary, whereas lack of homozygosity in affected individuals 13, 14, and 15 at marker D1S207 suggest the causal mutations lies proximal to marker D1S207. Taken together, this places the disease locus in a 18.38 cM (19.94 Mb) region on chromosome 1p31 flanked by D1S230 proximally and D1S207 distally. This critical interval harbors *RPE65*, which has been associated with autosomal recessive RP and LCA, previously. Bidirectional Sanger sequencing of *RPE65* identified a single base substitution in the splice acceptor site of intron 2; c.95–1G>A (Figure 4). This mutation is predicted to interrupt the open reading frame of RPE65 leading to a premature termination of the protein: p.R33Sfs11X. However, additional experimental evidence is required to rule out other plausible scenarios such as use of a cryptic splice site etc. This mutation segregated with the disease phenotype in the family and was not present in the 192 ethnically matched control chromosomes.

**Figure 5:**
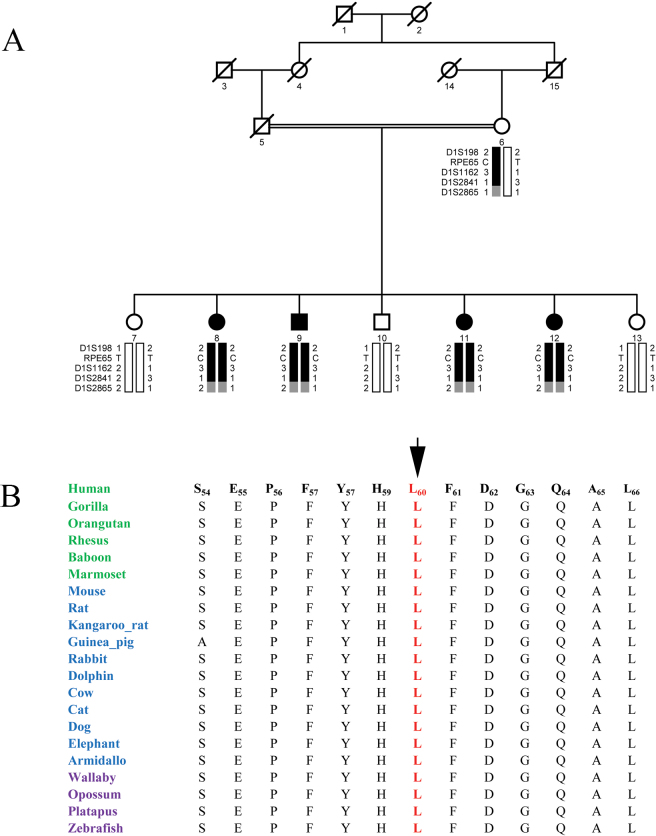
Pedigree drawing with sequence conservation of the L60 residue of *RPE65*. **A**: Pedigree drawing of family PKRP160 with haplotypes formed with chromosome 1p markers. Pedigree symbols are described in Figure 1. **B**: Sequence conservation of the L60 residue in other *RPE65* orthologs. Primates are green, placental mammals are blue, and vertebrates are purple. The arrow points to the amino acid residue L60 that is mutated in individuals affected with retinitis pigmentosa.

**Figure 6:**
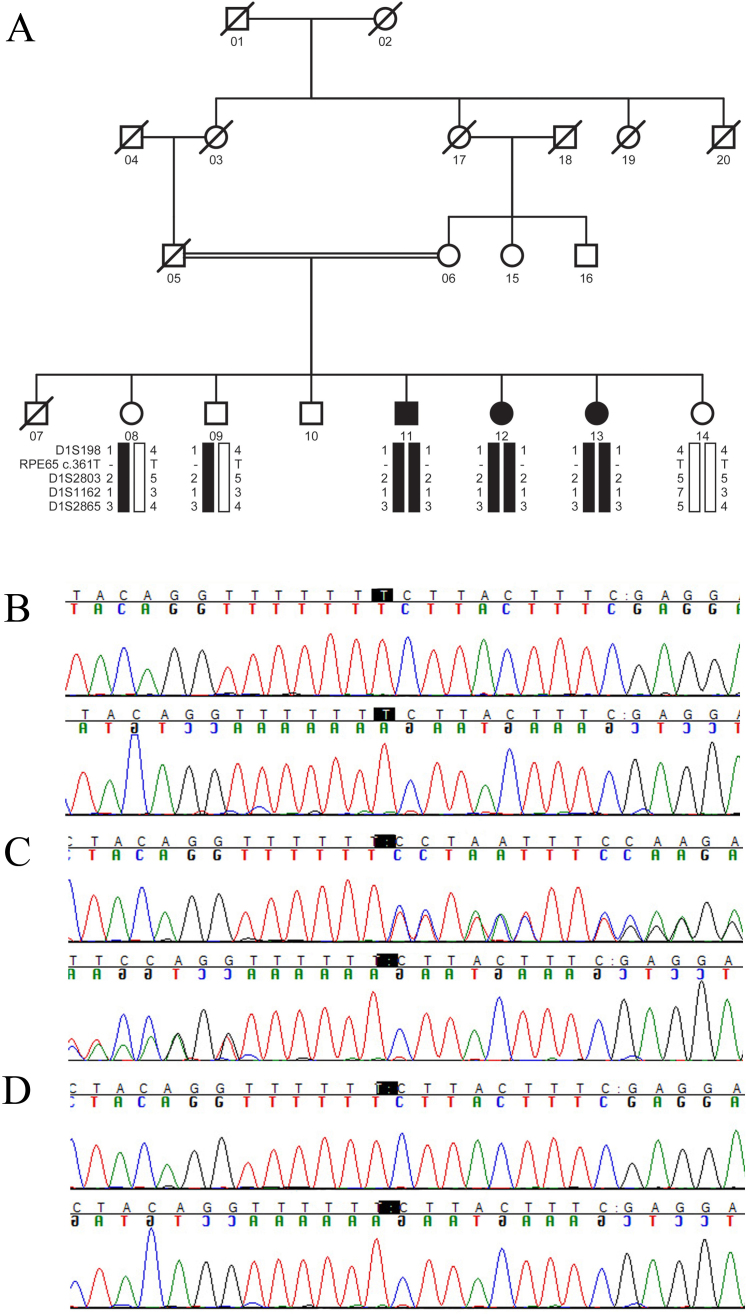
Pedigree drawing with bidirectional sequence chromatograms. **A**: Pedigree drawing of family PKRP235 with haplotypes formed using chromosome 1p markers. Pedigree symbols are described in Figure 1. **B**: Forward and reverse sequence chromatograms of unaffected individual 14 harboring the wild-type allele. **C**: unaffected individual 09, heterozygous carrier and, **D**: affected individual 11 homozygous for single base deletion: c.361delT.

Subsequently, we interrogated our entire cohort of small nuclear families with closely spaced fluorescently labeled STR markers spanning the chromosome 1p locus and identified two additional families, PKRP160 and PKRP235, in whom linkage and haplotype analyses suggested linkage to chromosome 1p (Figure 5 and Figure 6; Table 2B,C). The fundus examination of the affected individuals in family PKRP160 show bone spicules in the periphery of the fundus with retinal attenuation of the blood vessels (Figure 2C), whereas the rod and cone responses were not detected, suggesting an advanced stage of rod-cone dystrophy (Figure 3E–H).

Two-point parametric LOD scores of 1.97, 3.04, and 3.04 were obtained with markers D1S198, D1S1162, and D1S2841 at θ=0, respectively, for family PKRP160 (Table 2B). Haplotype analysis shows that all affected individuals of PKRP160 have homozygous alleles for markers D1S1162 and D1S2841 (Figure 5). Similarly, two-point parametric LOD scores of 2.50, 2.45, 2.47, and 2.39 were obtained with markers D1S198, D1S2803, D1S1162, and D1S2865 at θ=0, respectively, for family PKRP235 (Table 2C). Haplotype analysis shows that all affected individuals of PKRP235 have homozygous alleles for markers D1S198, D1S1162, D1S2841, and D1S2865 (Figure 6).

Sequencing of all coding exons of *RPE65* in PKRP160 identified a T to C transition in exon 3: c. 179T>C (Figure 4), which results in a leucine to proline substitution: p.L60P. All affected individuals were homozygous for single base substitution whereas the unaffected individuals were either heterozygous carriers or were homozygous for the wild-type allele. Additionally, this variation was not present in 192 ethnically matched control chromosomes. As shown in Figure 5, L60 is highly conserved in *RPE65* orthologs. Thus, we examined the possible impact of L60P substitution on the RPE65 protein with SIFT and PolyPhen. The SIFT algorithms predicted that L60P would not be tolerated by the native protein structure. Likewise, position-specific score differences obtained from PolyPhen suggested that L60P substitution could potentially have a deleterious effect on the RPE65 structure with position-specific independent counts (PSIC) score of 1.99 (a PSIC score difference >1.0 is probably damaging).

Finally, bidirectional sequencing of coding exons of *RPE65* in PKRP235 identified a single base deletion in exon 5: c. 361delT (Figure 6) that results in a frame shift leading to a premature termination of the open reading frame: p.S121Lfs*6. All affected individuals are homozygous for this deletion mutation whereas the unaffected individuals were either heterozygous carriers or were homozygous for the wild-type allele (Figure 6). Additionally, this variation was not present in 192 ethnically matched control chromosomes.

## Discussion

Herein, we report three consanguineous Pakistani families diagnosed with autosomal recessive RP. Genome-wide linkage and exclusion studies suggested linkage to chromosome 1p31 harboring the *RPE65* gene. Sequencing of *RPE65* identified three novel variations that segregated with the disease phenotype in the respective families and were not present in 192 ethnically matched control chromosomes. Linkage to chromosome 1p31 harboring the *RPE65* gene, segregation of causal variants with the disease phenotype, and their absence in ethnically matched controls strongly suggest that these variations are responsible for the disease phenotype. Identification of these mutations reaffirms the diverse allelic heterogeneity of *RPE65* in the pathogenesis of retinal dystrophies.

The mutations present in PKRP020 and PKRP235 are predicted to produce an unstable transcript that will be degraded by nonsense mediated decay [23,24]. If somehow the mutant messenger RNA escapes nonsense mediated decay, the protein thus produced will lack 502 and 412 amino acids of the C-terminal domain in affected individuals of PKRP020 harboring the splice acceptor mutation and affected individuals of PKRP0235 harboring a single base deletion. The evolutionary conservation of amino acid L60 in other RPE65 orthologs and PSIC score differences obtained from PolyPhen suggest that p.L60P has a deleterious effect on the RPE65 structure; however, the mechanism of the pathogenesis remains elusive. Identification of pathogenic mutations in *RPE65* will help in elucidating the structure-function relationship of the RPE65 protein, which will lead to development of novel therapeutic approaches.
